# Probiotic *Lactobacillus johnsonii* BS15 Improves Blood Parameters Related to Immunity in Broilers Experimentally Infected with Subclinical Necrotic Enteritis

**DOI:** 10.3389/fmicb.2018.00049

**Published:** 2018-01-30

**Authors:** Hesong Wang, Xueqin Ni, Xiaodan Qing, Lei Liu, Jinge Xin, Min Luo, Abdul Khalique, Yan Dan, Kangcheng Pan, Bo Jing, Dong Zeng

**Affiliations:** ^1^Animal Microecology Institute, College of Veterinary, Sichuan Agricultural University, Chengdu, China; ^2^Chongqing Fisheries Science Research Institute, Chongqing, China

**Keywords:** subclinical necrotic enteritis, *Lactobacillus johnsonii*, serum immunity, probiotic, broiler

## Abstract

The probiotic strain *Lactobacillus johnsonii* BS15 could exert beneficial effects on growth performance, lipid metabolism, and intestinal microflora in healthy broilers and those afflicted with subclinical necrotic enteritis (SNE). In particular, BS15 prevents SNE by enhancing intestinal immunity. To further understand the immune regulatory mechanism of BS15, we evaluated its effects on the overall immunity of broilers by determining blood parameters in healthy and SNE broilers. In this study, two experiments were conducted. Experiment 1 involved a 42-day experimental period and used 450 1-day-old male chicks. The chicks were randomly divided into three groups and fed with a basal diet with or without 1 × 10^5^ or 10^6^ colony-forming units (cfu) BS15/g as feed. Experiment 2 involved a 28-day experimental period and used 180 1-day-old male chicks. The chicks were randomly allotted into three groups and given with or without 1 × 10^6^ cfu BS15/g BS15 as feed. SNE infection was treated in all broilers, except in those in the normal diet group. Antioxidant abilities, immunoglobulins, and cytokines in the serum were assessed. T-lymphocyte subsets in peripheral blood were also determined. The first experiment demonstrated that BS15 enhanced the antioxidant abilities; the serum levels of immunoglobulins, interleukin-2, and interferon-gamma; and CD3^+^CD4^+^ T-lymphocyte percentage in peripheral blood on day 21. However, limited significant changes were observed on day 42. The second experiment revealed that BS15 supplementation positively influenced the antioxidant abilities and increased the serum levels of immunoglobulins and cytokines that were affected by SNE. BS15 also positively affected T-lymphocyte subsets in peripheral blood during SNE infection. These findings suggest that BS15 supplementation may prevent SNE in broilers by improving blood parameters related to immunity and enhancing intestinal immunity. Furthermore, BS15 supplementation can improve blood parameters in healthy broilers, especially at the starter phase.

## Introduction

Prohibiting the use of antibiotic growth promoters worldwide has led to serious problems in animal production, including decreased growth performance and breakout of diseases that are originally controlled by antibiotics (Darabighane and Nahashon, 2014; Wielinga et al., 2014; Zou et al., 2016). The ban on antibiotic growth promoters also led to necrotic enteritis (NE), which is one of the most threatening diseases of poultry and is mainly caused by *Clostridium perfringens* (Songer and Meer, 1996; Mcdevitt et al., 2006). Subclincal NE (SNE) presents without obvious clinical symptoms and is characterized by poor growth performance without mortality, resulting in huge economic losses (Olkowski et al., 2008; Shojadoost et al., 2012). In this regard, alternatives to antibiotic growth promoters for preventing SNE in broilers must be developed. Probiotics are live cultures of beneficial bacteria that exert beneficial effects on the host and are considered one of the most potent candidates for preventing the emergence of SNE in broilers (Sleator, 2010; Khani et al., 2012).

*Lactobacillus johnsonii* BS15 (CCTCC M2013663) is a probiotic strain that was first isolated from homemade yogurt collected from Hongyuan Prairie, Aba Autonomous Prefecture, China and reported to prevent non-alcoholic fatty liver diseases in obese mice (Xin et al., 2014). In our previous study, we found that incorporating BS15 to broiler diet enhanced growth performance and improved meat quality (Liu et al., 2016). Moreover, only live BS15 and not disrupted cells may exert health benefits mainly by improving lipid metabolism, intestinal development, and gut microflora in the small intestine (Wang et al., 2017c). We then applied BS15 to prevent SNE and discovered that it may positively control the growth performance, lipid deposition, and fatty acid composition of chicken meat during SNE infection by enhancing intestinal development and balancing microflora in the intestines (Qing et al., 2017; Wang et al., 2017a).

Various probiotic strains, even of the same bacterial species, may exhibit relatively different properties and clinical effects or exert the same influence under different mechanisms (Jacobsen et al., 1999; Servin and Coconnier, 2003). Therefore, the mechanism through which probiotics affect the host is difficult to elucidate. Researchers suggest that probiotic strains may exert their beneficial effects via different mechanisms and involve other microbiota (Lin et al., 2017). Aside from regulating the gut microbiota and improving lipid and/or energy metabolism, probiotics can enhance immunity and thus benefit the host (Shen et al., 2014; Zhou et al., 2016). However, limited information is available regarding the relationship between BS15 and immune responses in broilers. Our previous study showed that BS15 supplementation may prevent SNE-affected growth decline mainly by enhancing intestinal immunity in broilers (Wang et al., 2017b). Based on the results of two previous experiments (Liu et al., 2016; Qing et al., 2017), the present study was conducted to demonstrate the relationship between the beneficial effects of BS15 on body immunity. The effects of dietary BS15 supplementation on blood parameters related to immunity were assessed in healthy and SNE-afflicted broilers.

## Materials and Methods

### Feed Preparation

Viable counts of BS15 cell preparations were evaluated by heterotrophic plate counts after maintaining cultures in de Man, Rogosa, and Sharpe (MRS) broth at 37°C for 36 h under an anaerobic environment. Supplementation was performed before each feeding. The basic procedure was as follows: approximately 10 mL (1 mL in low dose BS15 group) of BS15 solution/MRS liquid medium (diluted with the same amount of phosphate buffer saline) was thoroughly mixed with 1000 g of diet. The number of viable bacteria altered over time in the diet was shown in our recent study (Wang et al., 2017c).

### Animals and Treatment

Experiment 1: A total of 450 one-day-old male chicks (Cobb 500) with similar body weight were assigned to 3 treatment groups consisting of 6 replicates with 25 birds per replicate. The three groups of birds were fed from days 1 to 42 as follows: control group (basal diet), L-BS15 group (basal diet + 1 × 10^5^ cfu BS15/g as feed), and H-BS15 group (basal diet + 1 × 10^6^ cfu BS15/g as feed).

Experiment 2: A total of 180 one-day-old male chicks (Cobb 500) with similar body weights were weighed and divided into three treatment groups. Each group consisted of six replicates with 10 birds per replicate. Three bird groups were fed with diets in mash form as follows: control group [basal diet + MRS liquid medium (normal diet)], SNE group (normal diet) and BS15 group [basal diet + 1 × 10^6^ colony-forming units (cfu) BS15/g as fed]. All chicks in the SNE and BS15 groups were gavaged orally with 20,000 *Eimeria acervulina* oocysts and 5,000 *E. maxima* oocysts (both of them are field isolates) per bird on 15 days of age and then with 1 mL of CP (2.2 × 10^8^ cfu/ml) from days 18–22 for SNE induction, whereas the control group was gavaged with 1 ml of PBS on 15 days of age and days 18–22 (Qing et al., 2017).

The birds were purchased from Chia Tai broiler hatchery (Chengdu, China) and the animal experiments were undertaken at the Key Laboratory of Animal Disease and Human Health of Sichuan Province, Sichuan Agricultural University. The diet formula is shown in **Table 1**. All diets were formulated to meet or exceed the National Research Council [NRC] (1994) requirements for broilers. Birds were fed *ad libitum* and given free access to water throughout the entire experiment. The temperature of the room was maintained at 33°C for the first 3 days, after which the temperature was gradually reduced by 3°C a week until it reached 24°C, where it was maintained for the remainder of the experiment. Artificial light was provided 24 h/day using fluorescent lights. All animal experiments were performed in accordance with guidelines for the care and use of laboratory animals and approved by the Institutional Animal Care and Use Committee of Sichuan Agricultural University (approval number: SYXKchuan2014-187).

**Table 1:** Composition of basal diets for broiler chickens.

Ingredient^1^	Starter diet (%) 1–21 days	Finisher diet (%) 22–42 days^3^
Ground yellow corn	56.00	59.50
Soybean meal	37.00	32.90
Soybean oil	3.66	4.70
Ground limestone	0.57	0.50
Dicalcium phosphate	1.80	1.60
Salt	0.30	0.30
Choline chloride	0.10	0.10
DL-Met	0.24	0.12
Micronutrients^2^	0.33	0.33
**Calculated nutrients level**		
ME (MJ kg^-1^)	12.40	12.80
CP	21.20	19.70
Lys	1.19	1.08
Met	0.50	0.40
Met + Cys	0.86	0.74
Ca	0.85	0.77
Nonphytate P	0.44	0.40

*^*1*^The ingredient and nutrient composition are reported on an as-fed basis. ^*2*^Micronutrients were provided per kilogram of diet: vitamin A (all-trans retinol acetate), 12,500 IU; cholecalciferol, 2,500 IU; vitamin E (all-rac-a-tocopherol acetate), 18.75 IU; vitamin K (menadione Na bisulfate), 5.0 mg; thiamine (thiamine mononitrate), 2.5 mg; riboflavin, 7.5 mg; vitamin B6, 5.0 mg; vitamin B12, 0.0025 mg; pantothenate, 15 mg; niacin, 50 mg; folic acid, 1.25 mg; biotin, 0.12 mg; Cu (CuSO_*4*_⋅5H_*2*_O), 10 mg; Mn (MnSO_*4*_⋅H_*2*_O), 100 mg; Zn (ZnSO_*4*_⋅7H_*2*_O), 100 mg; Fe (FeSO_*4*_⋅7H_*2*_O), 100 mg; I (KI), 0.4 mg; and Se (Na_*2*_SeO_*3*_), 0.2 mg. ^*3*^Birds was fed with finisher diet from days 22 to 28 in the second experiment.*

### Sampling

On the mornings of days 21 and 42 of Experiment 1 and days 28 of Experiment 2, six birds (one bird per cage) of each treatment were randomly selected. At least 10 mL per bird of peripheral blood samples from the wing vein were collected using anticoagulation tube. At least 2 mL per bird of serum samples were obtained from these blood samples by incubation at 4°C for 30 min and subsequent centrifugation at 1,500 × *g* for 20 min and serum samples were preserved at -20°C before assay.

### Antioxidant Indices, Immunoglobulins and Cytokines

Levels of immunoglobulins, including immunoglobulin G (IgG), IgM, and IgA, as well as cytokines including interleukin (IL)-2, IL-4, IL-6, tumor necrosis factor-alpha (TNF-α), and interferon-gamma (IFN-γ) in the serum were quantified using enzyme-linked immunosorbent assay (ELISA) kits specific for chicken (R&D Systems, Minneapolis, MN, United States) following manufacturer’s instructions. Antioxidant indices in the serum were measured using commercial kits by Nanjing Jiancheng Bioengineering Institute (Nanjing, Jiangsu, China); these indices included total antioxidation capacity (T-AOC), activities of catalase (CAT), superoxide dismutase (SOD), glutathione peroxidase (GSH-Px), inhibition of hydroxy radical (IHR), and malondialdehyde (MDA) and GSH contents, in the serum were quantified using ELISA kits specific for chicken (R&D Systems, Minneapolis, MN, United States).

### T-Lymphocyte Subsets Detection

The peripheral blood of six birds (one bird per cage) of each treatment were taken to determine CD3^+^, CD3^+^CD4^+^, and CD3^+^CD8^+^ T-lymphocyte percentages by flow cytometry method, as described by Chen et al. (2009). One milliliter anti-clotting peripheral blood was put in a test tube containing 1 mL 0.1 mol/L (pH7.4) phosphate-buffered saline (PBS) and then transferred to centrifuge tube containing 2 mL lymphocyte separation medium (Solarbio, Beijing, China) and centrifuged at 200 × *g* for 20 min. Approximately 0.5 mL lymphocyte layer was collected, transferred to another centrifuge tube, and then 2 mL PBS added and centrifuged at 200 × *g* for 5 min. The supernatant was discarded. The cell concentration was determined using the normal counting method of blood cells and then diluted to 1.0 × 10^6^ cells/mL with PBS. The aforementioned 1 mL cell suspension was transferred to another centrifuge tube and centrifuged at 200 × *g* for 5 min. The supernatant was discarded. The cells were, respectively, stained with 10 μL mouse anti-chicken CD4-phyto-erythrin (BD Pharmingen, United States) and mouse anti-chicken CD8a-FITC (BD Pharmingen) for 15–20 min at RT, and then 2 mL PBS added and centrifugal elutriation performed once. The supernatant was discarded. The cells were resuspended in 0.5 mL PBS and determined by fluorescence-activated cell sorter (BD Pharmingen) (Wu et al., 2012).

### Statistical Analysis

Data are expressed as means ± standard deviation and analyzed by one-way ANOVA. Duncan’s multiple-range test was used for multiple comparison when variances were not homogeneous. All the statistical analyses were conducted using SigmaPlot for Social Sciences version 12. Differences at *P* < 0.05 were considered statistically significant. Data were based on individual broilers (six replicates of one chick per cage).

## Results

### Antioxidant Indices

**FIGURE 1:**
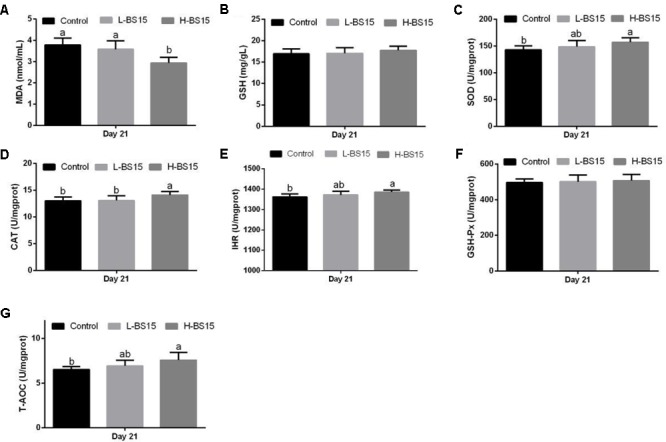
Changes of MDA **(A)**, GSH **(B)**, SOD **(C)**, CAT **(D)**, IHR **(E)**, GSH-Px **(F)**, and T-AOC **(G)** in the serum at days 21 in Experiment 1. Bars with different letters are significantly different on the basis of Duncan’s multiple range tests (*P* < 0.05). Data are presented as mean ± standard deviation (six replicates of one chick per cage). T-AOC, total antioxidation capacity; CAT, catalase; SOD, superoxide dismutase; GSH-Px, glutathione peroxidase; IHR, inhibiting hydroxy radical; MDA, malondialdehyde; GSH, glutathione.

**FIGURE 2:**
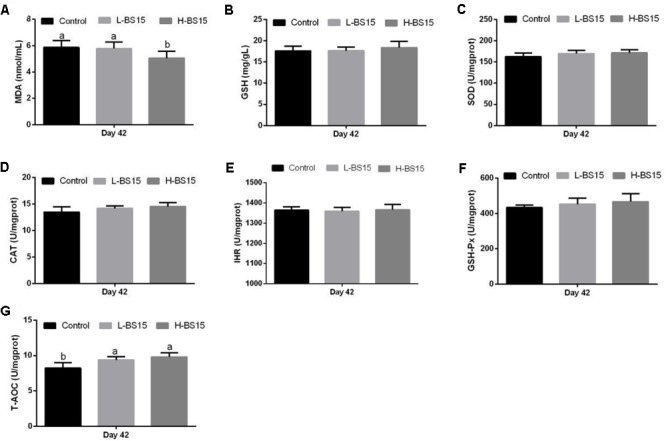
Changes of MDA **(A)**, GSH **(B)**, SOD **(C)**, CAT **(D)**, IHR **(E)**, GSH-Px **(F)**, and T-AOC **(G)** in the serum at days 42 in Experiment 1. Bars with different letters are significantly different on the basis of Duncan’s multiple range tests (*P* < 0.05). Data are presented as mean ± standard deviation (six replicates of one chick per cage). T-AOC, total antioxidation capacity; CAT, catalase; SOD, superoxide dismutase; GSH-Px, glutathione peroxidase; IHR, inhibiting hydroxy radical; MDA, malondialdehyde; GSH, glutathione.

**FIGURE 3:**
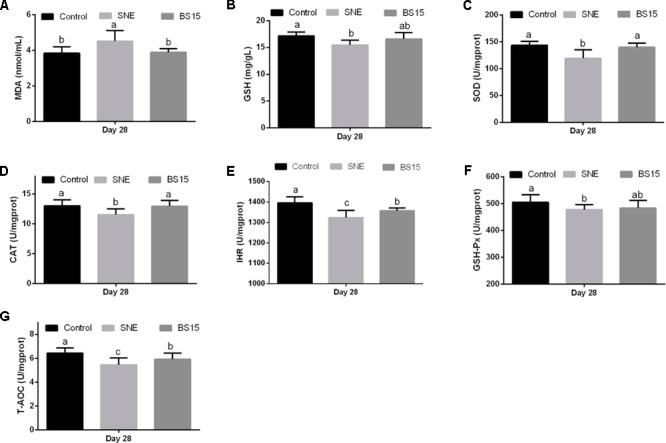
Changes of MDA **(A)**, GSH **(B)**, SOD **(C)**, CAT **(D)**, IHR **(E)**, GSH-Px **(F)**, and T-AOC **(G)** in the serum at days 28 in Experiment 2. Bars with different letters are significantly different on the basis of Duncan’s multiple range tests (*P* < 0.05). Data are presented as mean ± standard deviation (six replicates of one chick per cage). T-AOC, total antioxidation capacity; CAT, catalase; SOD, superoxide dismutase; GSH-Px, glutathione peroxidase; IHR, inhibiting hydroxy radical; MDA, malondialdehyde; GSH, glutathione.

The serum antioxidant indices in healthy and SNE-afflicted broilers are presented in **Figures 1, 2**, respectively (Experiment 1). **Figure 1** shows that the H-BS15 group exhibited significantly lower MDA levels (**Figure 1A**) (*P* < 0.05) but significantly higher SOD, CAT, IHR, and T-AOC (**Figures 1C–E,G**) (*P* < 0.05) than those in the control group on day 21. No significant changes in GSH content and GSH-Px activity were observed (**Figures 1B,F**) on day 21. On day 42, MDA level (**Figure 2A**) was lower (*P* < 0.05) in the H-BS15 group, and T-AOC (**Figure 2G**) was higher (*P* < 0.05) in both L-BS15 and H-BS15 compared with the control group; the other indices remained unchanged on day 42 (**Figures 2B–F**). **Figure 3** illustrates the results on BS15 use to prevent SNE (Experiment 2). The levels of serum antioxidant indices (**Figures 3A–G**) were significantly decreased (*P* < 0.05) in the SNE group compared with those in the control group, whereas those of SOD, CAT, IHR, and T-AOC (**Figures 3C–E,G**) were significantly higher (*P* < 0.05) in the BS15 group than those in the SNE group.

### Immunoglobulin Levels

**FIGURE 4:**
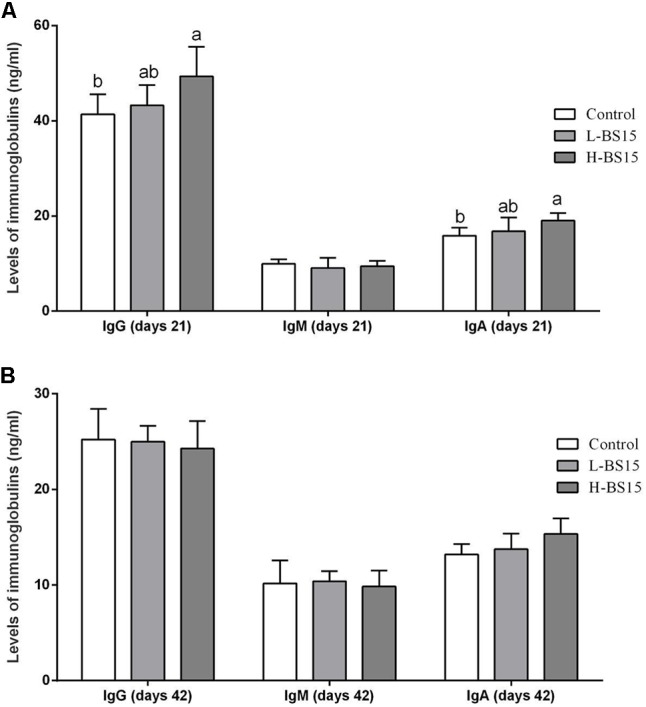
Levels of immunoglobulins in the serum (Experiment 1). Bars with different letters are significantly different on the basis of Duncan’s multiple range tests (*P* < 0.05). Data are presented as mean ± standard deviation (six replicates of one chick per cage). **(A)** Changes of IgG, IgM, and IgA in the serum at days 21; **(B)** changes of IgG, IgM, and IgA in the serum at days 42.

**FIGURE 5:**
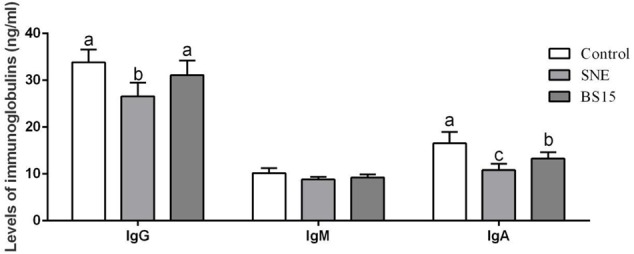
Levels of immunoglobulins in the serum (Experiment 2). Bars with different letters are significantly different on the basis of Duncan’s multiple range tests (*P* < 0.05). Data are presented as mean ± standard deviation (six replicates of one chick per cage).

The serum immunoglobulin levels of broilers were determined in Experiments 1 and 2 and are summarized in **Figures 4, 5**, respectively. As shown in **Figure 4A**, high BS15 supplement dose significantly increased IgG and IgA levels in the serum on day 21 compared with those in the control group (*P* < 0.05), but no significant difference was noted in IgM levels. The L-BS15 group exhibited no significant changes in immunoglobulin levels on day 21 (**Figure 4B**). **Figure 5** demonstrates that although no significant change was observed on IgM level (*P* > 0.05), the SNE group showed lower IgG and IgA levels than those in the control group (*P* < 0.05). Moreover, IgG and IgA levels in the BS15 group were significantly increased compared with those in the SNE group (*P* < 0.05).

### T-Lymphocyte Subsets

**FIGURE 6:**
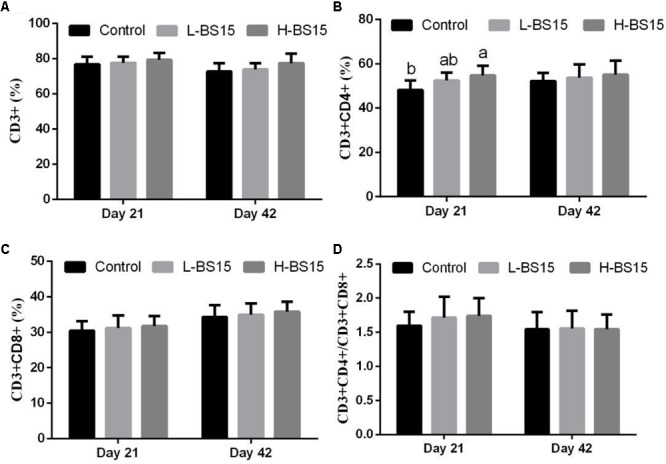
CD3^+^ **(A)**, CD3^+^CD4^+^ **(B)**, CD3^+^CD8^+^ **(C)**, T-lymphocyte percentages and CD3^+^CD4^+^/CD3^+^CD8^+^ ratio **(D)** in the peripheral blood at days 21 and 42 (Experiment 1). Bars with different letters are significantly different on the basis of Duncan’s multiple range tests (*P* < 0.05). Data are presented as mean ± standard deviation (six replicates of one chick per cage).

**FIGURE 7:**
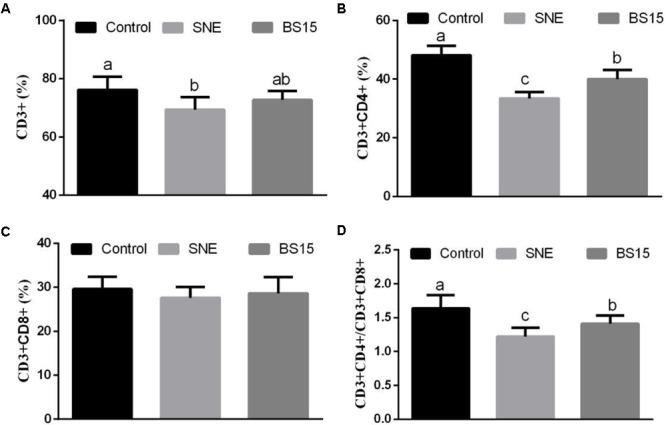
CD3^+^ **(A)**, CD3^+^CD4^+^ **(B)**, CD3^+^CD8^+^ **(C)**, T-lymphocyte percentages and CD3^+^CD4^+^/CD3^+^CD8^+^ ratio **(D)** in the peripheral blood (Experiment 2). Bars with different letters are significantly different on the basis of Duncan’s multiple range tests (*P* < 0.05). Data are presented as mean ± standard deviation (six replicates of one chick per cage).

**Figure 6** summarizes the T-lymphocyte subsets in the peripheral blood determined by FCM using different doses of BS15 in healthy broilers (Experiment 1). On days 21 and 42, no significant changes in all parameters (**Figures 6A,C,D**) were observed except on CD3^+^CD4^+^ T-lymphocyte percentage (*P* > 0.05, **Figure 6B**), which was significantly higher (*P* < 0.05) in the H-BS15 group than that in the control group. **Figure 7** presents the changes in peripheral blood T-lymphocyte subsets after BS15 supplementation against SNE, as determined in Experiment 2. As shown in **Figure 7A**, the CD3^+^ T-lymphocyte percentage was lower (*P* < 0.05) in the SNE group than that in the control group. CD3^+^CD4^+^ T-lymphocyte percentage (**Figure 7B**) and CD3^+^CD4^+^/CD3^+^CD8^+^ ratio (**Figure 7D**) in the BS15 group were higher (*P* < 0.05) than those in the SNE group but lower (*P* < 0.05) than those in the control group. However, no changes in the CD3^+^CD8^+^ T-lymphocyte percentage (**Figure 7C**) were detected among the three groups (*P* > 0.05).

### Levels of Cytokines

**FIGURE 8:**
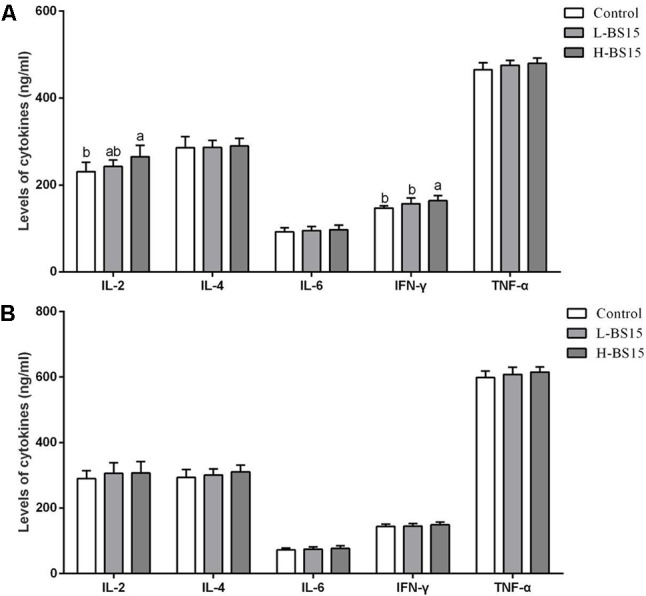
Levels of cytokines in the serum at days 21 and 42 (Experiment 1). Bars with different letters are significantly different on the basis of Duncan’s multiple range tests (*P* < 0.05). Data are presented as mean ± standard deviation (six replicates of one chick per cage). **(A)** Changes of IL-2, IL-4, IL-6, INF-γ, and TNF-α in the serum at days 21; **(B)** changes of IL-2, IL-4, IL-6, INF-γ, and TNF-α in the serum at days 42. TNF-α tumor necrosis factor-alpha; INF-γ interferon-gamma.

**FIGURE 9:**
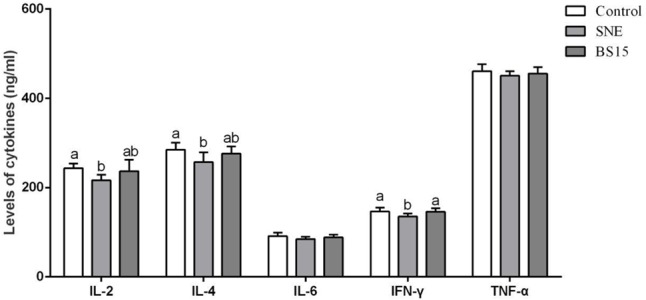
Levels of cytokines in the serum (Experiment 2). Bars with different letters are significantly different on the basis of Duncan’s multiple range tests (*P* < 0.05). Data are presented as mean ± standard deviation (six replicates of one chick per cage). TNF-α tumor necrosis factor-alpha; INF-γ interferon-gamma.

Serum cytokine levels in Experiment 1 are summarized in **Figure 8**. As shown in **Figures 8A,B**, IL-2 and IFN-γ levels were higher in the H-BS15 group than those in the control group (*P* < 0.05). However, no significant changes were detected in other cytokines, as illustrated in **Figure 8** (*P* > 0.05). **Figure 9** reveals the serum cytokine levels of broilers provided with BS15 to prevent SNE (Experiment 2). Although IL-6 and TNF-α levels did not change (*P* > 0.05), IL-2, IL-4, and IFN-γ levels were significantly lower (*P* < 0.05) in the SNE group compared with those in the control group. The IFN-γ level in the BS15 group was also higher (*P* < 0.05) than that in the SNE group.

## Discussion

The susceptibility of broilers to stress is a major problem in modern intensive poultry industry. Birds are often subjected to stressors, such as fasting, transport, and exposure to high or low environmental temperature (Eid et al., 2006). Stress causes declined feed consumption, growth rate, feed efficiency, fertility, and meat quality (El-Lethey et al., 2000; Avanzo et al., 2001). Himmelfarb (2004) reported a link between oxidative stress and inflammation in kidney disease. Oxidative stress can also induce molecular lesions and thus trigger apoptosis (Kasprzak, 1991). Therefore, antioxidant ability is crucial for the health and growth performance of broilers. The results in Experiment 1 revealed that the levels of T-AOC, MDA, SOD, CAT, and IHR indices were positively influenced by BS15, especially on day 21. MDA is one of the several low-molecular-weight end products formed via the decomposition of certain primary and secondary lipid peroxidation products (Janero, 1990). MDA production alters membrane fluidity and increases membrane fragility (Chen and Yu, 1994). SOD and CAT are antioxidant enzymes that are considered to be the first line of cellular defense against oxidative damage (Ferreccio et al., 1998). Our results indicated that BS15 supplementation could enhance the antioxidant ability of health broilers, especially at the starter phase. Consistent with our results, the findings of Shen et al. (2014) showed that serum antioxidant abilities are significantly enhanced while using *L. plantarum* to promote growth performance in broilers. Aluwong et al. (2013) also observed that yeast probiotic can increase body weight and enhance the serum anti-oxidant enzyme activities of broiler chickens. Aside from the abovementioned antioxidant indices, GSH also plays a primary role and is regarded as an early biological marker of oxidative stress among non-enzymatic antioxidants (Gagliano et al., 2006). Moreover, GSH-Px is an important antioxidant enzyme, which plays a particularly important role in antioxidant protection of the cell by the conversion of hydrogen peroxides to less harmful components (Olafsdottir and Reed, 1988). In Experiment 2, all of the determined indices related to oxidative damage are negatively correlated with SNE, which is in agreement with previous studies reporting a link between NE and oxidative stress parameters in the serum of chickens (Lovasova et al., 2009). However, differences in most indices were not significant between the BS15 and control group, suggesting that BS15 could enhance the antioxidant ability of broilers while SNE occurs. Previous research works on the antioxidant properties of probiotics also showed that probiotic strains that can prevent and control several diseases may contribute in limiting excessive amounts of reactive radicals *in vivo* (Spyropoulos et al., 2011; Amaretti et al., 2012).

Immunoglobulins, which play an important role in immune regulation and mucosal defense, can be influenced by various conditions and stimulations (Zhao et al., 2013). IgY is the major type of antibodies produced by chickens and is functionally equivalent to IgG in birds, reptiles, and amphibians (Gan et al., 2015). IgG mediates a wide range of functions, including transplacental passage and opsonization of antigens, which occurs through the binding of antigen-antibody complexes to specialized Fc receptors on macrophages and other cell types (Luo et al., 2013). IgM regulates subsequent immune response development, accelerating the production of high-affinity IgG and providing initial response against foreign antigens (Ehrenstein et al., 2000). IgA is critical for protecting mucosal surfaces against toxins, viruses, and bacteria by neutralizing or preventing these pathogens from binding to mucosal surface. IgA is also an important serum immunoglobulin, mediating a variety of protective functions through interaction with specific receptors and immune mediators (Bienenstock et al., 1973). Probiotics promote growth performance in different animals by increasing immunoglobulin levels. For example, the addition of a *Bacillus*-based probiotic as a growth promoter was found to increase IgG levels in the plasma of calves (Riddell et al., 2008). Experiment 1 revealed increased IgG and IgA on day 21, suggesting the ability of dietary BS15 to enhance immunity in the host. The changes in immunoglobulins in our study were in agreement with the results reported by Yalçin et al. (2016), which showed that probiotics linearly increased serum IgG concentration and decreased serum cholesterol concentration in broilers. Meanwhile, BS15 could control SNE by enhancing immunoglobulins levels as the IgA and IgG in the BS15 group were significantly higher than those in the SNE group. However, limited research studies demonstrate the changes in serum immunoglobulins during SNE infection.

T-lymphocyte subsets in peripheral blood are one of the most important indicators of overall immunity level. T-lymphocytes can be further classified based on their expression of cell surface proteins, specifically CD3—the molecular surface marker of mature T cells. CD3^+^ T cells form T cell receptors/CD3 ligation in signal transmission and then activate T cells. According to their cell surface identification, mature T cells can be divided into two main subsets, namely, CD4^+^ and CD8^+^ (Spangler et al., 2015). CD3^+^CD4^+^ T cells are associated with major histocompatibility complex (MHC) class II molecules and act as helper or inflammatory T cells in response to exogenous antigens, whereas CD3^+^CD8^+^ T cells are associated with MHC class I molecules and play a crucial role as cytotoxic T cells in response to endogenous antigens (Brisbin et al., 2008). CD3^+^CD4^+^/CD3^+^CD8^+^ ratio is considered as a direct index for evaluating the condition of body immunity (Stüve et al., 2006). Brisbin et al. (2012) reported that *Lactobacillus* species can regulate intestinal T lymphocyte subsets and that these responses may be important to intestinal homeostasis. In Experiment 1, no significant change was detected except for CD3^+^CD4^+^ T-lymphocyte percentage, which was higher in the H-BS15 group than the control group, suggesting a limited influence of BS15 on healthy broilers. However, remarkable changes were observed in Experiment 2, revealing that SNE significantly decreased CD3^+^ and CD3^+^CD4^+^ T-lymphocyte percentages and CD3^+^CD4^+^/CD3^+^CD8^+^ ratio in the peripheral blood. Moreover, CD3^+^CD4^+^ T-lymphocyte percentage and CD3^+^CD4^+^/CD3^+^CD8^+^ ratio in the peripheral blood were higher in the BS15 group than those in the SNE group, suggesting that BS15 can exert beneficial effects on the T-lymphocyte subsets and thus prevent SNE. As IgA and IgG levels, which are decreased by SNE in the serum, were also significantly increased in BS15 group in the present study, BS15 might prevent SNE through both T- and B-lymphocytes.

As determinants and modulators of immune pathology, cytokines play a key regulatory role among the many components of the animal immune system (Lin and Karin, 2007). Cytokine production is largely dependent on the differentiation state of T-cells, which can be divided into two different types according to the pattern of cytokine production (Deng et al., 2013). Among the cytokines determined in the present study, IL-2, TNF-α, and IFN-γ are produced by T helper 1 cells and play an important role in cell-mediated immune response. By contrast, IL-4 and IL-6 are secreted by T helper 2 cells and enhance humoral immunity (Kikuchi and Crystal, 2001). High doses of dietary BS15 improved the levels of IL-2 and IFN on day 21 in Experiment 1. IL-2, IFN, and IL-6 levels were lower in the SNE group than in the controls and enhanced by BS15. The results suggested that BS15 could stimulate the host to produce IL-2 and IFN-γ and thereby regulate cell-mediated immune response at the starter phase. The results also demonstrated the preventive effects of BS15 on cell-mediated immune response and humoral immunity induced by SNE. Furthermore, previous studies revealed that changes in T-lymphocyte subsets can alter cytokine levels to improve immunity, which was in agreement with our present results (Dearman et al., 1996).

We found that BS15 supplementation may prevent SNE-affected growth decline mainly by enhancing intestinal immunity in broilers. In the previous study, IgG and IgA levels in the ileum were decreased by SNE infection, and T-lymphocyte percentages in the lamina propria lymphocytes of ileum were decreased in SNE group. In addition, decreased antioxidant capacity and negatively influenced cytokine levels in the ileum were also observed while SNE occurred (Wang et al., 2017b). All of the mentioned changes are consistent with our present results on blood parameters, indicating that SNE may affect the whole organism through blood after eliciting intestinal immunity. In addition, gene expression in spleen is influenced by NE (Sarson et al., 2009), and *C. perfringens*-associated hepatitis occurs in NE broilers (Lovland and Kaldhusdal, 2001). The changes in blood parameters demonstrated in this study may partly explain the reason of liver and spleen damages caused by NE.

The results on growth performance, including daily weight gain, feed intake, and feed conversion ratio, are discussed in our recent articles, demonstrating that BS15 can significantly protect birds from decreased growth performance due to SNE (Qing et al., 2017). Although slight changes were observed on the growth performance after providing different doses of BS15 (Liu et al., 2016), our present study showed that high doses of BS15 significantly increased the starter, finisher, and overall daily weight gain. Our recent study also indicated that these effects on growth performance may be related to digestive ability and lipid metabolism altered by BS15 (Wang et al., 2017c). Probiotics may promote host growth by manipulating the microbial composition and gut immune responses of the host (Vieira et al., 2013; Salzman, 2014). Beneficial microbes cooperate with immunity to provide colonization resistance to pathogens and thus prevent gut diseases (Sassone-Corsi and Raffatellu, 2015). In the present study, BS15 significantly enhanced blood parameters related to immunity in both healthy and SNE-afflicted broilers, which provided evidence for the relationship between the beneficial effects of BS15 and its ability on immunological enhancement. Notably, BS15 exerted better effects while preventing SNE compared with its application in healthy broilers as more blood parameters were significantly changed by BS15 in the SNE group compared with those in the control group (Experiment 2). Most of recent studies on probiotics attribute the mechanism to the direct or indirect regulation of gut microbiota (Kalliomäki et al., 2008). Lin et al. (2017) reported that NE can disrupt the cecal microbiota of chickens, which may be alleviated through *Bacillus licheniformis* supplementation and thereby control the disease. We also observed the same recently reported beneficial effects of BS15 on the gut microbiota of healthy and SNE-afflicted broilers (Qing et al., 2017; Wang et al., 2017c). Based on the present study, we hypothesize that BS15 could help the host to balance its gut microbiota, which could be protective once intestinal diseases occur. Moreover, according to the results shown in Experiment 1, most of the blood parameters were changed on day 21, which proved our recent report that BS15 tended to confer benefits during the starter phase of production (Wang et al., 2017c).

## Conclusion

The feed supplementation with *L. johnsonii* BS15 may enhance blood parameters related to immunity, which may be one of the mechanisms of its beneficial effects in healthy and SNE-afflicted broilers.

## Author Contributions

HW, DZ, and XN designed the experiments. LL, KP, XQ, JX, and BJ performed the experiments. LL, YD, ML, and AK analyzed the experimental data. HW and DZ wrote this paper. All authors read and approved the final manuscript.

## Conflict of Interest Statement

The authors declare that the research was conducted in the absence of any commercial or financial relationships that could be construed as a potential conflict of interest.
